# Pathogenesis of Multiple Organ Injury in COVID-19 and Potential Therapeutic Strategies

**DOI:** 10.3389/fphys.2021.593223

**Published:** 2021-01-28

**Authors:** Miquéias Lopes-Pacheco, Pedro Leme Silva, Fernanda Ferreira Cruz, Denise Battaglini, Chiara Robba, Paolo Pelosi, Marcelo Marcos Morales, Celso Caruso Neves, Patricia Rieken Macedo Rocco

**Affiliations:** ^1^Biosystems & Integrative Sciences Institute, Faculty of Sciences, University of Lisbon, Lisbon, Portugal; ^2^Laboratory of Pulmonary Investigation, Carlos Chagas Filho Biophysics Institute, Federal University of Rio de Janeiro, Rio de Janeiro, Brazil; ^3^National Institute of Science and Technology for Regenerative Medicine, Rio de Janeiro, Brazil; ^4^Rio de Janeiro Innovation Network in Nanosystems for Health-NanoSAÚDE/FAPERJ, Rio de Janeiro, Brazil; ^5^COVID-19 Virus Network, Ministry of Science, Technology and Innovation, Brasília, Brazil; ^6^COVID-19 Virus Network, Brazilian Council for Scientific and Technological Development, Brasília, Brazil; ^7^COVID-19 Virus Network, Fundação de Amparo à Pesquisa do Estado do Rio de Janeiro – FAPERJ, Rio de Janeiro, Brazil; ^8^Anesthesia and Intensive Care, San Martino Policlinico Hospital, IRCCS for Oncology and Neuroscience, Genoa, Italy; ^9^Department of Surgical Sciences and Integrated Diagnostic, University of Genoa, Genoa, Italy; ^10^Laboratory of Cellular and Molecular Physiology, Carlos Chagas Filho Biophysics Institute, Federal University of Rio de Janeiro, Rio de Janeiro, Brazil; ^11^Laboratory of Biochemistry and Cell Signaling, Carlos Chagas Filho Institute of Biophysics, Federal University of Rio de Janeiro, Rio de Janeiro, Brazil

**Keywords:** ACE2, coronavirus, lung, multiple organ dysfunction, pathophysiology, SARS-CoV-2, therapy, viral infection

## Abstract

Severe acute respiratory disease coronavirus 2 (SARS-CoV-2, formerly 2019-nCoV) is a novel coronavirus that has rapidly disseminated worldwide, causing the coronavirus disease 2019 (COVID-19) pandemic. As of January 6th, 2021, there were over 86 million global confirmed cases, and the disease has claimed over 1.87 million lives (a ∼2.2% case fatality rate). SARS-CoV-2 is able to infect human cells by binding its spike (S) protein to angiotensin-conversing enzyme 2 (ACE2), which is expressed abundantly in several cell types and tissues. ACE2 has extensive biological activities as a component of the renin-angiotensin-aldosterone system (RAAS) and plays a pivotal role as counter-regulator of angiotensin II (Ang II) activity by converting the latter to Ang (1-7). Virion binding to ACE2 for host cell entry leads to internalization of both via endocytosis, as well as activation of ADAM17/TACE, resulting in downregulation of ACE2 and loss of its protective actions in the lungs and other organs. Although COVID-19 was initially described as a purely respiratory disease, it is now known that infected individuals can rapidly progress to a multiple organ dysfunction syndrome. In fact, all human structures that express ACE2 are susceptible to SARS-CoV-2 infection and/or to the downstream effects of reduced ACE2 levels, namely systemic inflammation and injury. In this review, we aim to summarize the major features of SARS-CoV-2 biology and the current understanding of COVID-19 pathogenesis, as well as its clinical repercussions in the lung, heart, kidney, bowel, liver, and brain. We also highlight potential therapeutic targets and current global efforts to identify safe and effective therapies against this life-threatening condition.

## Introduction

In late 2019, a cluster of infections by a novel coronavirus – the severe acute respiratory syndrome coronavirus 2 (SARS-CoV-2, formerly 2019-nCoV) – was epidemiologically linked to a large seafood market in Wuhan, China (Wang C. et al., 2020; Zhou P. et al., 2020). This outbreak has rapidly disseminated domestically and internationally, becoming a global pandemic^1^. SARS-CoV-2 has demonstrated to be much more infectious than SARS-CoV and Middle East Respiratory Syndrome coronavirus (MERS-CoV), which were responsible for two previous large-scale outbreaks (Table 1). As of January 6th, 2020, there were over 86 million global confirmed cases of coronavirus disease 2019 (COVID-19) and over 1.87 million fatalities^2^ ^3^.

**TABLE 1:** Main features of SARS, MERS, and COVID-19.

Disease/Outbreak	SARS (2002)	MERS (2012)	COVID-19 (2019)
***Virology***			
Etiological agent	SARS-CoV	MERS-CoV	SARS-CoV-2
Genome size (kb)	30.1	27.9	29.9
Natural reservoir	Bats	Bats	Bats
Intermediate host	Civet cats/Raccoon dogs	Camels	Pangolins (?)
Attachment receptor	ACE2	DPP4	ACE2
Spike protein priming	TMPRSS2	–	TMPRSS2
Basic reproduction number (R_0_)	2.2-3.7	0.3-1.3	2.2-6.4
***Epidemiology***			
First cases	Guangdong, China	Jeddah, Saudi Arabia	Wuhan, China
Main route of transmission	Airborne	Airborne	Airborne
Incubation period (days after infection)	2-10	2-15	2-14
Peak viral load (days after symptom onset)	∼10	7-10	3-7
Hospitalization rate	Most cases	Most cases	∼20%
Cases	8,096	2,494	> 86,000,000*
Deaths (case fatality rate)	744 (9.2%)	858 (34%)	>1,870,000 (∼2.2%)*
***Clinical features and management***			
Common symptoms	Fever, dry cough, dyspnea, shortness of breath, fatigue	Fever, dry cough, dyspnea, shortness of breath, fatigue	Fever, dry cough, dyspnea, shortness of breath, fatigue
Common laboratory findings	Abnormalities in coagulation and blood cell counts, cytokine storm, increased levels of transaminases and C-reactive protein	Abnormalities in coagulation and blood cell counts, cytokine storm, increased levels of transaminases and C-reactive protein	Abnormalities in coagulation and blood cell counts, cytokine storm, increased levels of transaminases and C-reactive protein
Chest computed tomography findings	Multiple, focal, ground-glass opacities, atelectasis or bilateral patchy consolidations in lungs	Multiple, focal, ground-glass opacities, atelectasis or bilateral patchy consolidations in lungs	Multiple, focal, ground-glass opacities, atelectasis or bilateral patchy consolidations in lungs
Complications	ARDS, renal failure, sepsis or septic shock	ARDS, renal failure, sepsis or septic shock	ARDS, renal failure, sepsis or septic shock
Therapeutic approach	Early intensive care and supportive monitoring	Early intensive care and supportive monitoring	Early intensive care and supportive monitoring
Vaccine or specific antiviral available	No	No	Multiple vaccines are receiving interim or conditional approval for emergency use in different countries (refer to Table 2)

*As of January 6th, 2021.

**FIGURE 1:**
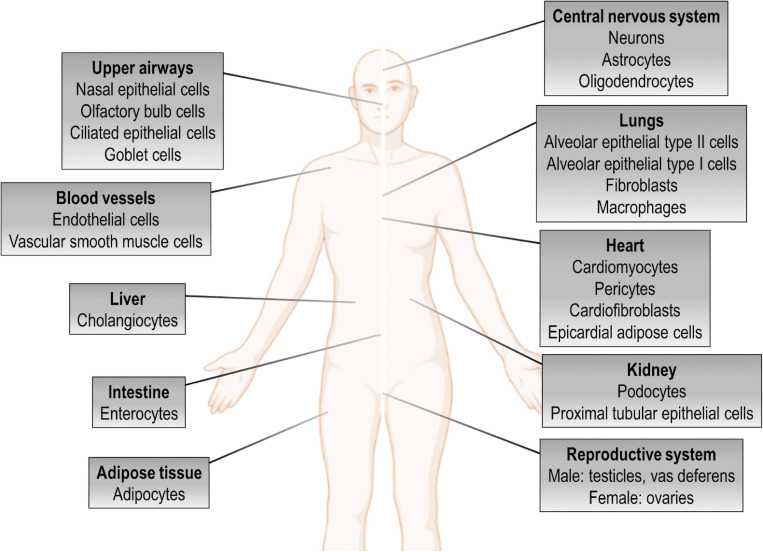
ACE2 expression in the human body. Tissue distribution of angiotensin-converting enzyme 2 (ACE2) expression and potential targets for direct cytotoxicity induced by SARS-CoV-2 infection.

Human coronaviruses usually attach to cell-surface ectopeptidases, such as dipeptidyl peptidase 4 (DPP4), aminopeptidase N, and angiotensin-conversing enzyme 2 (ACE2), for host cell entry (Fehr and Perlman, 2015; Cui et al., 2019). Both SARS-CoV and SARS-CoV-2 were found to use ACE2, which has extensive biological activities (Santos et al., 2018), as their key cell entry receptor (Hoffmann et al., 2020a; Walls et al., 2020; Zhou P. et al., 2020). The distribution of ACE2 in the host organism appears to be an important factor associated with organ injury (Figure 1). There is also evidence that individual variation in expression and/or polymorphisms in ACE2 may influence susceptibility to SARS-CoV-2 infection and COVID-19 phenotype (Calcagnile et al., 2020; Cao et al., 2020a; Chen J. et al., 2020; Devaux et al., 2020; Hou et al., 2020; Hussain et al., 2020; Leung et al., 2020).

The clinical spectrum of COVID-19 is very heterogeneous (Williamson et al., 2020). Many individuals infected with SARS-CoV-2 are asymptomatic or develop a mild illness with non-specific symptoms, such as fever, fatigue, dry cough, and headache (Huang C. et al., 2020; Sakurai et al., 2020; Wang C. et al., 2020). However, ∼20% of individuals require hospitalization, and ∼25% of these (∼5% of all cases) experience a rapid progression of their symptoms to severe pneumonia/acute respiratory distress syndrome (ARDS), requiring invasive mechanical ventilation (Guan et al., 2020; Huang C. et al., 2020; Wang D. et al., 2020). Individuals with a more severe phenotype usually have a high viral load and long virus-shedding period (He et al., 2020; Liu et al., 2020b). Some risk factors for the development of severe COVID-19 and poor prognosis include advanced age and presence of certain comorbidities, such as chronic obstructive pulmonary disease, coronary heart disease, diabetes mellitus, and hypertension (Guan et al., 2020; Leung et al., 2020; Wu and McGoogan, 2020; Williamson et al., 2020; Zhou F. et al., 2020), although the latter remains controversial (Iaccarino et al., 2020). Multivariable parameters such as higher sequential organ failure assessment score and D-dimer >1 μg/mL on hospital admission have also been associated with higher risk of fatality (Chen N. et al., 2020; Guan et al., 2020; Tang et al., 2020; Zhou F. et al., 2020).

Although SARS-CoV-2 infection was initially described as causing severe respiratory disease, it is now known that infected individuals can rapidly progress to a multiple organ dysfunction syndrome (MODS); therefore, multi-target therapeutic approaches are warranted, and a wide range of distinct therapeutic protocols have been investigated (Gupta et al., 2020; Li H. et al., 2020b; Robba et al., 2020a, b; Zhang et al., 2020b). In this review, we summarize major features of SARS-CoV-2 biology and the current understanding of COVID-19 pathogenesis, as well as its clinical repercussions in the lung, heart, kidney, bowel, liver, and brain. We also shed light on potential therapeutic targets and the current global efforts to identify effective therapies against this devastating condition.

## Structure of SARS-CoV-2 and Host Cell Infection

### The SARS-CoV-2 Genome

**FIGURE 2:**
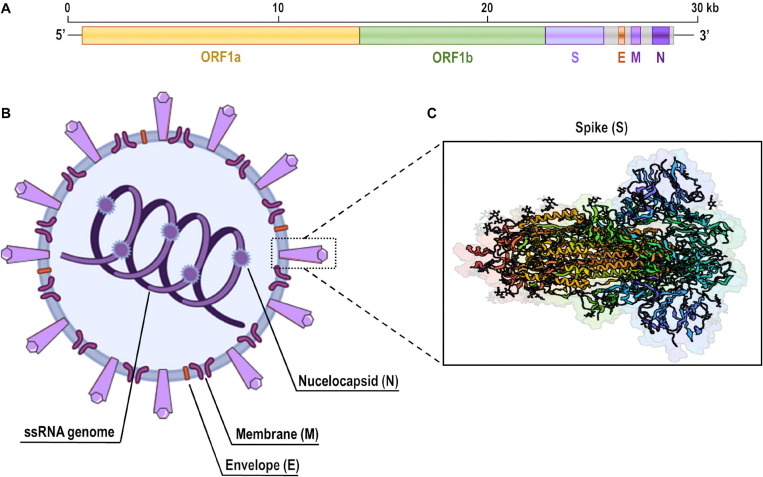
Genome and structure of SARS-CoV-2. **(A)** Full-length single-strand (ss)RNA genome of SARS-CoV-2 demonstrating open-reading frames (ORFs) 1a and 1b, which encode non-structural proteins, and the position of genes that encode main structural proteins: spike (S), envelope (E), membrane (M), and nucleocapsid (N). **(B)** Schematic representation of a SARS-CoV-2 viral particle. **(C)** Ribbon diagram of the open conformation of SARS-CoV-2 spike protein. This structure has been deposited on the Protein Data Bank under accession number 6VYB (SARS-CoV-2 spike ectodomain structure).

Coronaviruses are named for the crown-shaped spikes on their outer surface. The novel coronavirus (SARS-CoV-2) is an enveloped, 29.9 kb-long, positive-sense, single-stranded RNA virus belonging to the β-coronavirus genus (Figure 2A). The open-reading frames (ORFs) 1a and 1b represent ∼70% of the complete viral genome and possess several conserved non-structural protein sequences (Chan et al., 2020; Kim et al., 2020; Wu A. et al., 2020). A frameshift between these ORFs encodes two polypeptides (1a and 1ab) that are processed by viral proteases to produce non-structural proteins, which are involved in viral replication and suppression of host innate immune defenses (Chan et al., 2020; Wu A. et al., 2020). Among all known coronavirus sequences, SARS-CoV-2 shares the highest genetic similarity with the bat coronavirus RATG13 (∼96%) and the Malayan pangolin coronavirus (∼91%), although it also has considerable genetic similarity with the human coronaviruses SARS-CoV (∼79%) and MERS-CoV (∼50%) (Andersen et al., 2020; Coronaviridae Study Group of the International Committee on Taxonomy of Viruses, 2020; Lam et al., 2020; Zhang T. et al., 2020).

### The SARS-CoV-2 Structure

The structure of SARS-CoV-2 is composed of four main structural proteins: the spike, envelope, and membrane glycoproteins, and the nucleocapsid protein (Figure 2B). The nucleocapsid is a phosphorylated protein which consists of the structure that directly binds to viral RNA and plays multiple critical roles during the viral life cycle. The envelope glycoprotein is a small, integral membrane structure involved in the maturation and pathogenesis of coronaviruses. The membrane glycoprotein is the most abundant component of the virus structure and plays a central role in viral assembly by interconnecting with all main structural proteins of the viral particle. This protein also delineates the shape of the viral envelope (Fehr and Perlman, 2015; Cui et al., 2019).

The spike glycoprotein is a transmembrane structure present in the outer surface of the viral particle (Figure 2C). It has two subunits (S1 and S2) that are cleaved by the host cell proteases. Interestingly, the furin protease-like cleavage spot is present in SARS-CoV-2, MERS-CoV, and human coronavirus OC43, but absent in SARS-CoV (Andersen et al., 2020; Coutard et al., 2020; Wrapp et al., 2020). The S1 subunit consists of an N-terminal domain and a receptor-binding domain (RBD), which determine host range and cellular tropism of the virus. This subunit is released during the fusion process, thus inducing a conformational change in the S2 subunit, which is the viral membrane-anchored fraction and consists of a hydrophobic fusion peptide and two heptad repeated domains (Cui et al., 2019; Wu A. et al., 2020).

### The Host Cell Receptor

**FIGURE 3:**
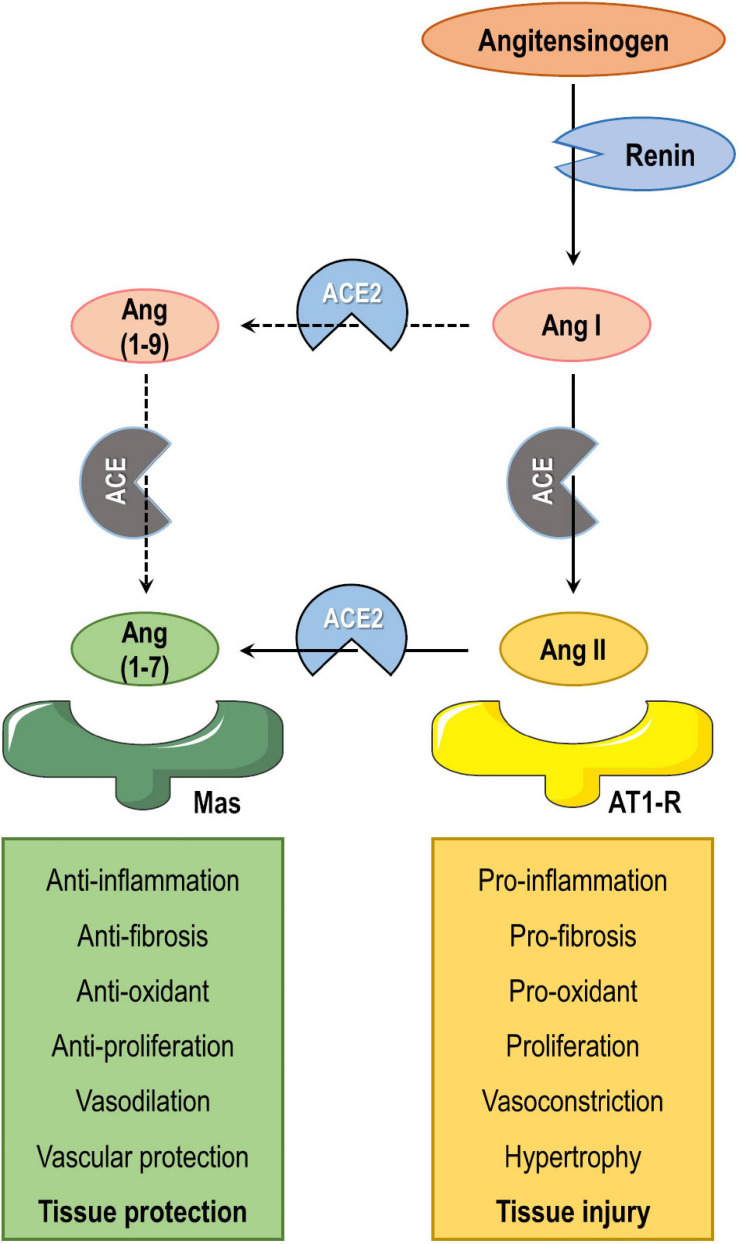
Simplified diagram of the renin-angiotensin-aldosterone system. Angiotensinogen is cleaved by renin to angiotensin I (Ang I). Ang I is then converted by angiotensin-converting enzyme (ACE) to Ang II, which interacts with the AT1 receptor (AT1-R), or is cleaved by ACE2 to Ang (1-7), which interacts with Mas receptor. The ACE2/Ang (1-7)/Mas axis exerts counter-regulatory effects to the ACE/Ang II/AT1-R axis in multiple organs. ACE2 can also convert Ang I to Ang (1-9), which is then cleaved to Ang (1-7) by ACE. However, ACE2 converts Ang II to Ang (1-7) with higher efficiency than it converts Ang I to Ang (1-9).

ACE2 is a zinc-dependent carboxypeptidase and component of the renin-angiotensin-aldosterone system (RAAS), a complex network that plays a pivotal role in maintaining fluid and electrolyte homeostasis, thus affecting function of multiple organs (Figure 3). Briefly, the protease renin cleaves angiotensinogen to angiotensin I (Ang I), which is subsequently cleaved to Ang II by ACE. Ang II can bind to angiotensin type 1 receptors (AT1-R) and induce vasoconstriction as well as pro-inflammatory, oxidant, and fibrotic responses (Forrester et al., 2018; Danser et al., 2020). On the other hand, ACE2 can convert Ang II to Ang (1-7), which binds to the Mas receptor and counteracts the aforementioned actions mediated by Ang II/AT1-R. ACE2 can also convert Ang I to Ang (1-9), which is subsequently cleaved to Ang (1-7) by ACE. However, the former pathway is more common due to higher affinity between ACE and Ang I (Santos et al., 2018; Gheblawi et al., 2020).

### SARS-CoV-2 Infection and Replication

**FIGURE 4:**
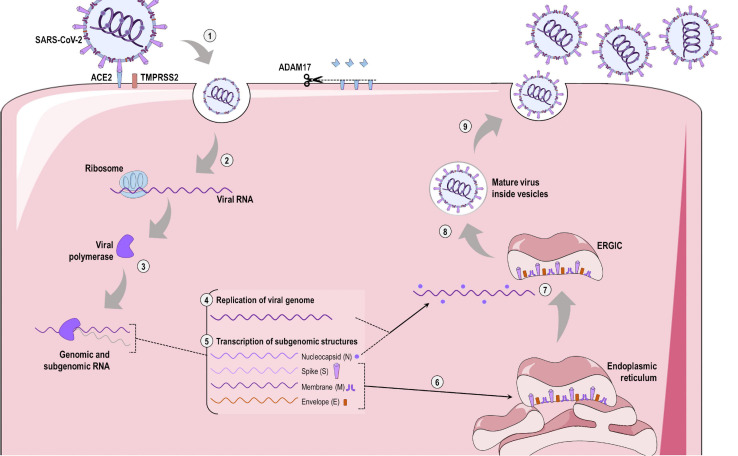
SARS-CoV-2 infection and replication. **(1)** SARS-CoV-2 spike protein is primed by the type II transmembrane serine protease (TMPRSS2) and interacts with the angiotensin-converting enzyme 2 (ACE2) expressed on the surface of host cells, allowing binding and entry of the virus via clathrin-dependent endocytosis. Concomitantly, ADAM17 is activated and leads to cleavage of ACE2 in its soluble form. **(2)** The SARS-CoV-2 virion uses host ribosomes to produce viral RNA-dependent RNA polymerase **(3)**. This viral polymerase then initiates replication of the SARS-CoV-2 genome **(4)** and transcription of subgenomic structures **(5)**. The spike, membrane, and envelope subgenomic transcripts use the endoplasmic reticulum for translation of these viral structural proteins **(6)**, while the viral genome joins the nucleocapsid. **(7)** All viral structures are exported to the endoplasmic-reticulum-Golgi intermediated compartment (ERGIC), where new viral particles are assembled. **(8)** After the formation of mature virions, these are exported inside Golgi vesicles to the extracellular compartment via exocytosis. **(9)** These new viruses are now able to infect adjacent ACE2-expressing cells or enter the bloodstream and infect other tissues.

SARS-CoV-2 RBD attaches to ACE2 for host cell entry (Figure 4), a proteolytic process that is facilitated by TMPRSS2 priming and involves cathepsin L (Hoffmann et al., 2020a; Ou et al., 2020; Wrapp et al., 2020; Zhou P. et al., 2020). This interaction then triggers endocytosis of ACE2 together with the SARS-CoV-2 virion and fusion of the viral membrane and host cell. Interestingly, SARS-CoV-2 not only reduces surface tissue expression of ACE2, but also inhibits its messenger RNA expression after infection (Kuba et al., 2005; Walls et al., 2020). Virus-host receptor interactions also activate ADAM17/TACE, a disintegrin and metalloprotease component, which leads to cleavage and shedding of ACE2 from the cell surface and local production of hyaluronan (Haga et al., 2010; Xu et al., 2017). Concomitantly, the viral spike protein is exposed to endosomal cysteine proteases that lead to its cleavage at two different sites: the first removes the S1 subunit, while the second occurs within the S2 subunit and results in exposure of the fusion peptide (Wan et al., 2020; Walls et al., 2020). Neutrophil elastase was recently found to exert an important role in SARS-CoV-2 infection, as it possesses a cleavage site near the S1-S2 subunits (Bhattacharyya et al., 2020). The viral package is released into the host cytoplasm, where it usurps the cellular machinery to produce new viral particles. As SARS-CoV-2 is a single-stranded RNA virus, its own genetic material serves as messenger RNA, thus driving the synthesis of viral proteins by host cell ribosomes. The uncoated viral RNA encodes the 1a and 1ab polypeptides, which are processed into non-structural proteins. These form a complex with viral genomic RNA to continuously synthesize subgenomic products that encode structural proteins (Jiang et al., 2020). TMPRSS2 also appears to participate in the SARS-CoV-2 replication process, although the mechanisms are unclear (Matsuyama et al., 2020). In fact, protein-protein mapping has identified 332 high-confidence SARS-CoV-2/human protein-protein interactions involved with the virus life cycle (Gordon D.E. et al., 2020). Once all viral components are produced and assembled in the endoplasmic reticulum–Golgi intermediate compartment (ERGIC), the new viruses are released via exocytosis into the extracellular compartment (Jiang et al., 2020).

## Pathogenesis and Multiple Organ Injury in COVID-19

**FIGURE 5:**
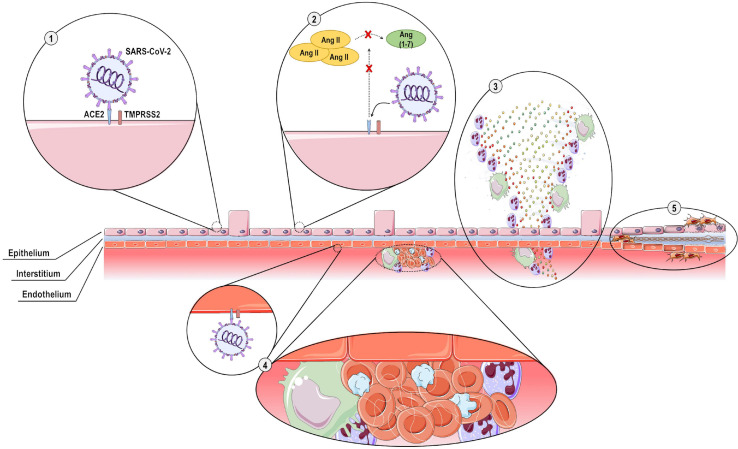
Pathogenesis of COVID-19. Five main pathological mechanisms are implicated in COVID-19 development and progression: **(1)** SARS-CoV-2 spike protein is primed by the type II transmembrane serine protease (TMPRSS2) and interacts with angiotensin-converting enzyme 2 (ACE2) expressed on the cell surface of epithelial cells, inducing direct cytotoxicity; **(2)** The interaction of SARS-CoV-2 with ACE2 causes downregulation of ACE2 expression, thus preventing cleavage of angiotensin II (Ang II) to Ang (1-7) and resulting in dysregulation of the renin-angiotensin-aldosterone system; **(3)** excessive acute inflammatory responses are elicited, with overproduction of pro-inflammatory cytokines and chemokines; **(4)** SARS-CoV-2 exerts direct cytotoxic effects on endothelial cells, leading to recruitment of pro-inflammatory cells and activation of the coagulation cascade, with intravascular thrombus formation; and **(5)** extensive tissue destruction with interstitial thickening, fibroblast proliferation, and fibrosis.

Many features of virus-host interactions involving SARS-CoV-2 remain unknown, but several of these have been demonstrated to recapitulate the infection process of other human coronaviruses. COVID-19 development and progression consist of five major pathological mechanisms (Figure 5): (1) direct virus-induced cytotoxicity in ACE2-expressing cells; (2) dysregulation of the RAAS as a result of virus-mediated ACE2 downregulation; (3) dysregulation of immune responses; (4) endothelial cell injury and thrombo-inflammation; and (5) tissue fibrosis (Gupta et al., 2020).

The lungs possess several features that facilitate their role as an initial reservoir for viral replication and human-to-human transmission. Lung tissue has a large surface area, making it highly susceptible to inhaled viruses. It is also highly vascularized, which allows rapid dissemination of viral particles to other organs. Furthermore, ACE2 is expressed in several pulmonary cell types (Hamming et al., 2004), with alveolar epithelial type II cells being the major ACE2-expressing cells (Zhao Y. et al., 2020). Gene ontology enrichment analysis has demonstrated that alveolar epithelial type II cells express multiple viral life cycle-associated functional genes, including those related to virus internalization, genome replication, assembly, and transmission (Zhao Y. et al., 2020).

The production and exocytosis of new viral particles can induce the host cell to undergo pyroptosis and release damage-associated molecular pattern signals. In lung tissue, these are recognized by adjacent epithelial and endothelial cells that are primed to activate certain transcription factors, such as nuclear factor-κB (NF-κB) and interferon regulatory factor 3 (IRF3), to produce and secrete pro-inflammatory mediators (Li G. et al., 2020). Concomitantly, secretion of type I interferons occurs and induces antiviral actions by multiple mechanisms. In an immunocompetent response, these initial inflammatory signals are recognized by antigen-presenting cells—such as resident macrophages and dendritic cells—that present the foreign antigen to CD4^+^ T-cells, which then prime other CD4^+^ T-cells, CD8^+^ T-cells, and B-cells (Channappanavar et al., 2014). CD8^+^ T-cells are cytotoxic and directly kill virus-infected cells, whereas B-cells produce neutralizing antibodies against the nucleocapsid and spike protein. Phagocytes remove apoptotic cells and neutralized viruses, resulting in well-coordinated clearance of infection with recovery and minimal lung tissue injury (Channappanavar et al., 2014; Li G. et al., 2020). On the other hand, during a faulty immune response occurs an intense inflammation with inefficient virus clearance, as well as overactivation of innate immunity with overproduction of pro-inflammatory cytokines and chemokines, including interleukin (IL)-1β, IL-6, tumor necrosis factor (TNF)-α, and others. This uncontrolled inflammatory process leads to alveolar-capillary barrier disruption; viral particles, along with the emerging cytokine storm, can then circulate to other organs, resulting in multiple organ dysfunction (Zhang et al., 2020b). Neutrophil extravasation into the alveolar space, hyaline membrane formation, and acute capillaritis are major features observed in histopathological analysis of lung cells in cases of severe COVID-19 (Barnes et al., 2020; Fox et al., 2020; Tian S. et al., 2020; BR236). The thromboembolic events observed in severe COVID-19 cases may also be associated with the inflammatory response triggered by the host-virus interaction, including activation of the coagulation cascade, formation of neutrophil extracellular traps (NETs), and leakage of fluid in the subendothelial compartment (Barnes et al., 2020; Helms et al., 2020; Tang et al., 2020; Varga et al., 2020).

**FIGURE 6:**
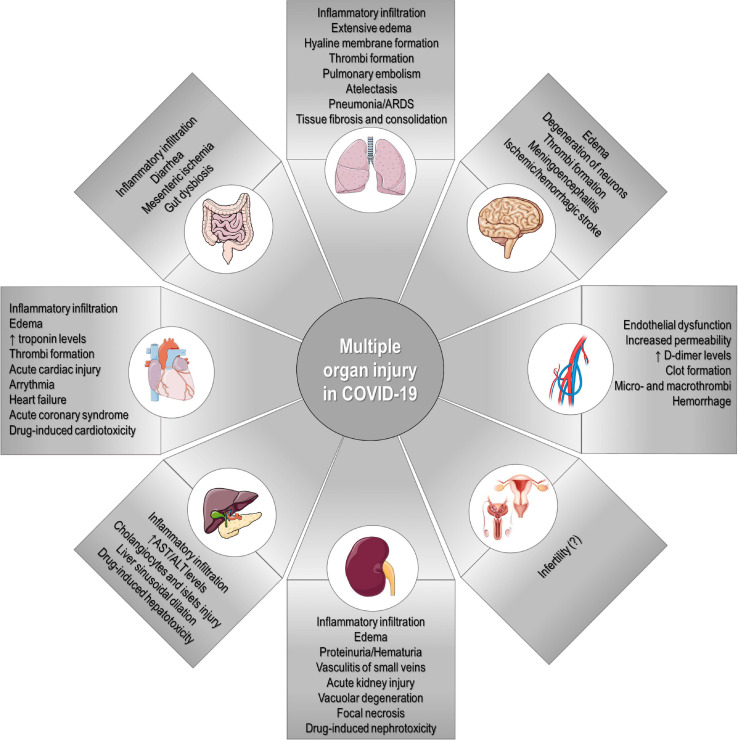
Multiple organ injury in COVID-19. SARS-CoV-2 infection can potentially cause multiple clinical manifestations and affect several organs, including the lungs, heart, blood vessels, kidney, intestine, liver, brain, and reproductive system.

The heterogeneous clinical manifestations in COVID-19 may be attributed to individual variation in expression not only of ACE2 and TMPRSS2 receptors, but also of genes related to inflammation and immune responses (Elhabyan et al., 2020; Ellinghaus et al., 2020; Russo et al., 2020; Zhao J. et al., 2020). Accordingly, the excessive acute inflammatory responses seen in individuals with severe COVID-19 may lead to MODS and death (Li H. et al., 2020b; Wang D. et al., 2020). Compared to healthy individuals, those infected with SARS-CoV-2 have demonstrated increased serum concentrations of many pro-inflammatory mediators, including IL-1β, IL-2, IL-6, IL-7, IL-8, interferon (IFN)-γ-induced protein 10 (IP-10), granulocyte colony-stimulating factor (G-CFS), monocyte chemoattractant protein 1 (MCP1), macrophage inflammatory protein (MIP)-1α, platelet-derived growth factor (PDGF), TNF-α, vascular endothelial growth factor (VEGF), and others (Ackermann et al., 2020; Chen N. et al., 2020; Huang C. et al., 2020; Liu J. et al., 2020; Zhang et al., 2020b). This cytokine storm is even more evident in individuals admitted to the intensive care unit (ICU), as demonstrated by greater increases in serum concentration of several of these pro-inflammatory mediators, which suggests a correlation with disease severity (Chen N. et al., 2020; Huang C. et al., 2020; Liu J. et al., 2020; Zhang et al., 2020b). Aberrant pathogenic CD4^+^ T-cells co-expressing IFN-γ and G-CFS have also been observed in severe COVID-19 (Zhou Y. et al., 2020). A high neutrophil-to-lymphocyte ratio has also been reported as a risk factor for mortality in hospitalized individuals with COVID-19 (Liu et al., 2020a). Nevertheless, individuals with severe COVID-19 may also present with lymphopenia, characterized by a significant reduction in peripheral CD4^+^ T-cell and CD8^+^ T-cell counts. Such countervailing effects may prevent complete clearance of SARS-CoV-2 or lead to secondary infections by opportunistic pathogens, thus resulting in a sepsis-like state (Guan et al., 2020; Liu J. et al., 2020; Zhou F. et al., 2020). Below, we summarize the main features of SARS-CoV-2 infection and its potential repercussions on multiple organs (Figure 6).

### Respiratory System

The lung is undoubtedly the organ most vulnerable to, and most affected by, SARS-CoV-2 infection. Accordingly, severe pneumonia is the most common and serious clinical manifestation observed in severe COVID-19 cases (Huang C. et al., 2020; Zhou Y. et al., 2020). As ACE2 is expressed in various cell types along the respiratory tract, SARS-CoV-2 may either enter through mucosal membranes in the upper respiratory tract or directly infect bronchial and alveolar epithelial cells in the lower respiratory tract (Sungnak et al., 2020; Wölfel et al., 2020). Interestingly, nasal expression of ACE2 was found to be lower in children compared to adults, which might partially explain age-related differences in the risk of developing COVID-19 (Bunyavanich et al., 2020; Sharif-Askari et al., 2020). SARS-CoV-2 RNA has also been detected in the sputum of infected individuals before the onset of clinical symptoms (Zhang W. et al., 2020), and in some cases, even 2 weeks after recovery (Lauer et al., 2020; Wölfel et al., 2020). Current and former smokers, as well as individuals with chronic obstructive pulmonary disease, overexpress ACE2 in airway cells compared to non-smokers and healthy individuals, which may explain at least partly the increased risk of severe COVID-19 in these individuals (Cai, 2020; Leung et al., 2020; Sharif-Askari et al., 2020).

ACE2 acts as a double-edged sword in both SARS-CoV and SARS-CoV-2 infection; it not only serves as the functional receptor for virus entry into host cells, but also has a pivotal role in protecting lung tissue from injury by counter-regulating the vasoconstrictive, pro-inflammatory, and pro-fibrotic effects of Ang II on pulmonary vascular and epithelial cells (Kuba et al., 2005; Uhal et al., 2011). Virus-receptor interactions lead to virion-ACE2 internalization (Wang et al., 2008) or cleavage and shedding of ACE2 (Haga et al., 2010; Xu et al., 2017), thus reducing its expression while promoting accumulation of Ang II, production of TNF-α and IL-6 receptor, and activation of macrophages to a pro-inflammatory state (Jia et al., 2009; Glowacha et al., 2010; Haga et al., 2010; Gheblawi et al., 2020). Furthermore, the virus nucleocapsid protein may interact with Smad3 to prevent apoptosis of infected host cells, while promoting transforming growth factor (TGF)-β-mediated tissue fibrosis (Zhao et al., 2008). As alveolar epithelial type II cells are self-renewing and express high levels of ACE2, they may be continuously targeted for viral entry and replication, which induces a vicious cycle of tissue injury and repair that may ultimately result in replacement of gas-exchange areas to non-functional fibrotic tissue. Lung stem/progenitor cells also express ACE2, and active virus replication in these cells may impair lung tissue repair (Ling et al., 2006).

In addition to the pivotal role of ACE2 in COVID-19 pathogenesis, a genome-wide association analysis identified other host genetic factors that may contribute to the development of COVID-19–induced respiratory failure. Although there were no single nucleotide polymorphism (SNP) associations in the human leukocyte antigen (HLA) complex, SNPs in a cluster of six genes on chromosome 3p21.31 (*SLC6A20, ZTFL1, CCR9, FYCO1, CXCR6, and XCR1*) were found to be potentially influential (Ellinghaus et al., 2020). Furthermore, ABO blood group has been suggested to be involved in susceptibility to SARS (Cheng et al., 2005a) and COVID-19 (Ellinghaus et al., 2020; Zhao J. et al., 2020; Zietz and Tatonetti, 2020).

Lung histopathological findings have demonstrated considerable similarities among ARDS, SARS, and COVID-19 (Tian S. et al., 2020; Xu Z. et al., 2020; Zhang H. et al., 2020). These consist of an increased neutrophil and mononuclear cell count, diffuse alveolar injury with proteinaceous alveolar exudates, and hyperplasia of epithelial type II cells. In more severe lung injury, thickened alveolar septa, hyaline membrane formation, and thrombus formation have been observed, as well as consolidation with fibroblast proliferation and fibrosis. Multinucleated giant cells in alveoli have also been found in some cases (Tian S. et al., 2020; Xu Z. et al., 2020; Zhang H. et al., 2020). An increased rate of deep venous thrombosis and pulmonary embolism was found in COVID-19 cases admitted to the ICU, despite prophylactic use of anticoagulation agents (Helms et al., 2020; Klok et al., 2020; Lodigiani et al., 2020); this can rapidly worsen lung function and lead to respiratory failure.

Based on chest computed tomography findings, COVID-19–induced lung impairment can be grouped into three main phenotypes: (1) multiple, focal, potently overperfused ground-glass opacities; (2) inhomogeneously dispersed atelectasis; and (3) typical moderate-to-severe ARDS, with alveolar edema and low compliance (Robba et al., 2020a). As these phenotypes may be related to different pathological mechanisms and disease progression, personalized mechanical ventilation approaches should be implemented in order to allow more efficient clinical recovery for each individual.

### Circulatory System

Coagulation dysfunction has been found in a high proportion of COVID-19 cases, as evidenced by increasing D-dimer levels and prolonged prothrombin time, as well as overt thrombotic manifestations (Guan et al., 2020; Helms et al., 2020; Huang C. et al., 2020; Tang et al., 2020; Wang D. et al., 2020). SARS-CoV-2 RNA has been detected in blood specimens of individuals with COVID-19 (Huang C. et al., 2020; Wölfel et al., 2020); as ACE2 is highly expressed in smooth muscle as well as arterial and venous endothelium in virtually all organs, these tissues can be directly targeted by SARS-CoV-2, with induction of endothelial dysfunction (Hamming et al., 2004; Ackermann et al., 2020; Varga et al., 2020). COVID-19–associated hypoxia resulting from pulmonary dysfunction leads to reduced blood flow and vasoconstriction, which also contribute to endothelial dysfunction (Helms et al., 2020; Levi et al., 2020; Varga et al., 2020). Furthermore, the excessive production of pro-inflammatory mediators leads to an imbalance between the amount of pro- vs. anti-coagulant factors and induction of platelet aggregation (Tang et al., 2020; Zuo et al., 2020). An increase in levels of thrombin, tissue factor V and VIII, and fibrinogen, alongside NET formation, results in a hypercoagulable state with increased risk of systemic macro- and micro-thrombosis (Barnes et al., 2020; Levi et al., 2020; Tang et al., 2020; Zuo et al., 2020). Indeed, fibrinous exudates and thrombi have been observed in histopathological specimens obtained from individuals with COVID-19 (Ackermann et al., 2020; Tian S. et al., 2020; Zhang H. et al., 2020). Therefore, laboratory monitoring of D-dimer levels, fibrinogen, platelet count and prothrombin time is crucial in all hospitalized and severe cases of COVID-19.

An increased incidence of cardiovascular complications has been observed in severe COVID-19, with a high incidence of clinical symptoms of heart disease (palpitations and chest tightness), elevated cardiac biomarker levels tests (especially cardiac troponin I or T), and abnormalities in electro- or echocardiography (Huang C. et al., 2020; Shi et al., 2020; Wang D. et al., 2020). In autopsy series of COVID-19 cases, most patients were found to have cardiomegaly, right ventricular dilatation (Fox et al., 2020), and mild fibrosis (Tian S. et al., 2020). The heart may be susceptible to direct SARS-CoV-2–mediated cytotoxicity, as ACE2 is highly expressed in cardiomyocytes (Hamming et al., 2004; Varga et al., 2020). Nevertheless, single-cell RNA sequencing has demonstrated that pericytes express higher levels of ACE2 than cardiomyocytes, and thus may also be targeted by SARS-CoV-2, leading to capillary endothelial dysfunction upon viral infection (Chen L. et al., 2020; Robba et al., 2020b). Heterozygosis for loss of ACE2 activity is believed to be sufficient to increase the risk of heart disease (Wang et al., 2014). The role of circulating soluble ACE2 is not well understood, but its levels are significantly increased in the presence of cardiovascular dysfunction and/or SARS-CoV-2 infection, and may be used as a biomarker (Úri et al., 2016; Xu et al., 2017; Sama et al., 2020; Wang K. et al., 2020). Furthermore, cytokine storm, compounded by a hypoxic state resulting from pulmonary dysfunction, may lead to direct myocardial injury. Early acute myocardial injury has been associated with a higher risk of in-hospital mortality in COVID-19 (Ni et al., 2020). Pre-existing cardiovascular diseases have also been associated with a worse prognosis, as COVID-19 may aggravate cardiac tissue injury and dysfunction (Chen N. et al., 2020; Wu and McGoogan, 2020; Zhou F. et al., 2020). Monitoring of cardiac function and standard biomarkers is recommended.

### Urogenital System

A balance between the effects of ACE2/Ang (1-7) and Ang II is tightly regulated to maintain normal kidney function. As ACE2 is expressed in kidney tissue, mainly in the brush border of the proximal cells, these cells are susceptible to direct injury caused by SARS-CoV-2 infection (Hamming et al., 2004; Fan C. et al., 2020). Laboratory tests have frequently revealed evidence of kidney dysfunction in individuals with COVID-19, including proteinuria, hematuria, and elevated levels of serum creatinine and blood urea nitrogen (Cheng et al., 2020; Hirsch et al., 2020; Li Z. et al., 2020). Renal abnormalities suggestive of inflammation and edema have also been observed on computed tomography (Li Z. et al., 2020). COVID-19–induced acute kidney injury has been associated with a markedly elevated risk of in-hospital mortality—approximately 5 times higher than that of COVID-19 patients without acute kidney injury (Chen N. et al., 2020; Cheng et al., 2020; Guan et al., 2020; Li Z. et al., 2020; Wang D. et al., 2020). A substantial number of individuals who develop COVID-19–induced acute kidney injury also require dialysis (Hirsch et al., 2020). SARS-CoV-2 viral antigens have been found in urine and in kidney tissue, mainly tubular epithelial cells and podocytes, at autopsy (Diao et al., 2020; Pan X. et al., 2020; Su et al., 2020); histopathological studies showed diffuse tubular injury with loss of brush border integrity, vacuolar degeneration, and necrosis (Su et al., 2020). Intraluminal erythrocyte aggregation in capillaries, without the presence of fibrinoid material, has also been reported (Su et al., 2020). The potential for direct viral damage to the kidney notwithstanding, inflammation and antiviral agents can also induce nephrotoxicity; therefore, kidney biomarkers and fluid and electrolyte status should be closely monitored to prevent kidney injury.

Despite a similar prevalence across genders, a higher case fatality rate has been observed in men with COVID-19, regardless of age (Jin et al., 2020). It is worth noting that testicular and vas deferens cells have a much higher expression of ACE2 than ovaries (Fan C. et al., 2020; Shastri et al., 2020; Wang and Xu, 2020), which could partially explain differences in disease severity and, consequently, fatality. Furthermore, sex-chromosome genes and sex hormones may contribute to the differential regulation of immunological responses between genders (Bukowska et al., 2017; Taneja, 2018). A high density of immune-related genes is located on the X chromosome, as is ACE2. This functional mosaic could influence innate and adaptive immune responses or disease severity (Taneja, 2018). In this context, women have been found to mount a more robust T-cell activation response than men during SARS-CoV-2 infection, which may contribute to a more efficient virus clearance (Takahashi et al., 2020).

### Gastrointestinal System

A considerable proportion of patients with COVID-19 present with abdominal pain, nausea, diarrhea, and vomiting, suggesting that SARS-CoV-2 infection may cause gastrointestinal dysfunction (Guan et al., 2020; Huang C. et al., 2020; Robba et al., 2020b; Wang D. et al., 2020; Zhou F. et al., 2020). Indeed, ACE2 is abundantly expressed in the luminal surface of intestinal epithelial cells (Hamming et al., 2004), where it acts as a co-receptor for amino acid uptake (Hashimoto et al., 2012). SARS-CoV-2 nucleocapsid protein has been found along multiple gastrointestinal structures (Lamers et al., 2020; Xiao et al., 2020), as well as in stool specimens (Holshue et al., 2020; Wölfel et al., 2020; Zhang et al., 2020a), which suggests that oral-fecal transmission might occur. Diffuse endothelial inflammation of the small intestine and mesenteric ischemia were observed in histopathological examination of COVID-19 cases (Varga et al., 2020). Although gastrointestinal symptoms have not been associated with increased risk of mortality, they appear to correlate with a longer duration of illness (Mao R. et al., 2020; Pan L. et al., 2020). Furthermore, altered liver function tests have been observed in several individuals with COVID-19, including elevated levels of transaminases (aspartate aminotransferase and alanine aminotransferase), total bilirubin (Guan et al., 2020; Huang C. et al., 2020; Wang D. et al., 2020), and γ-glutamyl transferase (Fan Z. et al., 2020; Zhang et al., 2020a). At autopsy, mild lobular infiltration by lymphocytes, central sinusoidal dilation, and patchy necrosis of the liver were reported in a case series of COVID-19 (Tian S. et al., 2020; Xu Z. et al., 2020). Kupffer cell proliferation and chronic hepatic congestion were also observed in another case series (Lax et al., 2020). As cholangiocytes express ACE2, they are susceptible to direct virus-induced cytotoxicity (Chai et al., 2020). However, it remains unclear whether liver injury in COVID-19 is due to SARS-CoV-2 infection, systemic inflammation, drug-related, or multifactorial. As most drugs under investigation as therapies for COVID-19 are metabolized in the liver, its function should be monitored periodically.

### Nervous System

Clinical manifestations consistent with neurological dysfunction, such as headache, confusion, and sudden loss of olfaction and gustation, as well as visual impairment, have been reported with increasing frequency in cases of COVID-19 (Butowt and Bilinska, 2020; Chen N. et al., 2020; Mao L. et al., 2020; Robba et al., 2020b; Wu P. et al., 2020). As ACE2 is expressed in olfactory bulb cells, neurons, astrocytes, and oligodendrocytes, the virus may rapidly disseminate through important brain areas once the olfactory epithelium is infected (Chen R. et al., 2020). SARS-CoV-2 may also infect the cerebral vascular endothelium and cross the blood–brain barrier via infected leukocytes, thus migrating into the central nervous system (Li Y. et al., 2020). Although no evidence of SARS-CoV-2 infection was observed in a first report of brain autopsies (Solomon et al., 2020), the presence of SARS-CoV-2 RNA in cerebrospinal fluid has been reported in patients with meningoencephalitis, suggesting that neurological manifestations might be due to direct invasion by SARS-CoV-2 (Huang Y.H. et al., 2020; Moriguchi et al., 2020). An autopsy series also reported cerebral edema and partial neuronal degeneration in individuals with COVID-19 (Xu Z. et al., 2020). Further evidence of SARS-CoV-2 neurotropism has been observed in other autopsies of COVID-19 cases, with such manifestations as lymphocytic encephalitis, meningitis, and massive intracranial hemorrhage (Von Weyhern et al., 2020). Furthermore, SARS-CoV-2 infection of the brainstem may be at least partially responsible for respiratory and cardiovascular failure (Li Y. et al., 2020).

## Potential Therapeutic Approaches

**FIGURE 7:**
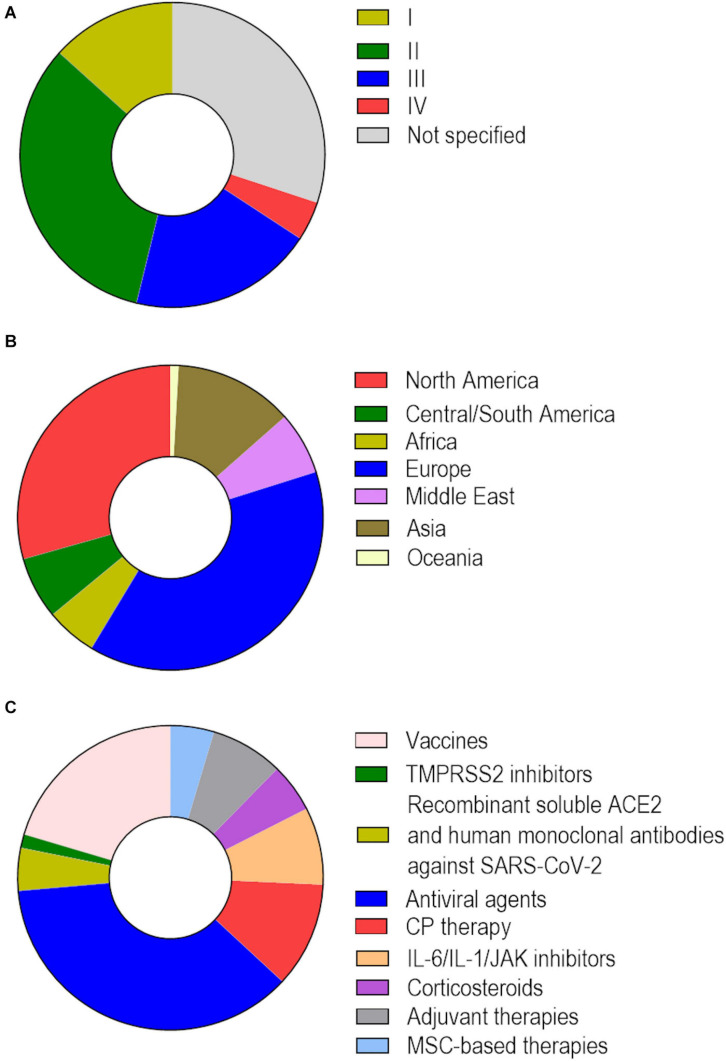
Clinical trials of COVID-19 therapeutics registered on the U.S. National Institutes of Health Clinical Trials Platform. As of December 7th, 2020, there were 4,094 registered clinical trials to evaluate the safety and efficacy of therapeutic approaches for COVID-19. **(A)** Distribution based on study phase: I (13.3%), II (32.9%), III (19.6%), and IV (4.1%). **(B)** Distribution based on region: North America (28.9%), Central/South America (6.5%), Africa (5.2%), Europe (37.9%), Middle East (6.5%), Asia (12.5%), Oceania (0.9%). **(C)** Distribution of 1,520 clinical trials based on therapeutic approaches under investigation: vaccines (20.4%), TMPRSS2 inhibitors (1.4%), recombinant soluble ACE2 and human monoclonal antibodies against SARS-CoV-2 (4.5%), agents with known or purported antiviral activity (ribavirin, favipiravir, remdesivir, lopinavir, umifenovir, ivermectin, nitazoxanide, chloroquine/hydroxychloroquine) (36.7%), convalescent plasma (CP) therapy (11.2%), IL-6/IL-1/JAK inhibitors (8.2%), corticosteroids (5.2%), adjuvant therapies (low-molecular-weight heparin, bevacizumab, recombinant human DNase) (7.7%), and mesenchymal stromal cell (MSC)-based therapies (4.6%).

The identification of similarities among human coronaviruses has provided some clues for the development of effective therapies (Table 1), especially for specific vaccines or effective antivirals against SARS-CoV-2. Several therapeutic approaches are under experimental and clinical investigation for COVID-19, but most are existing drugs approved for other disease indications. At least 66 human proteins or host factors can be targeted by clinically approved drugs in order to potentially inhibit SARS-CoV-2/host cell interactions (Gordon D.E. et al., 2020). Drug repurposing offers an advantage, as these drugs are already available in the clinic and have undergone extensive toxicological studies before marketing approval (Pushpakom et al., 2019; Battaglini et al., 2020; Lopes-Pacheco, 2020; Rocco et al., 2020). Therefore, the time frame to acquire an indication for COVID-19 may be reduced if safety and efficacy are demonstrated in late-stage clinical trials. Nevertheless, as COVID-19 is a multifaceted disease (Li H. et al., 2020b; Zhang B. et al., 2020b), multi-target therapeutic approaches are required to reduce or even prevent SARS-CoV-2 infection and its downstream effects—namely, systemic inflammation and multiple organ injury. To date, over 4,000 clinical trials of COVID-19 therapies have been registered in the U.S. National Institutes of Health Clinical Trials Platform^4^ (Figure 7). In this section, we collected a wide number of therapeutics under investigations and some that, despite being extensively evaluated in the clinic, demonstrated no efficacy against COVID-19.

### Vaccine Development

The best long-term strategy to stop SARS-CoV-2 transmission and prevent new infections is the development of an effective vaccine. There are many efforts in progress from both the scientific community and the pharmaceutical industry to develop vaccines against SARS-CoV-2; the current pipeline has over 200 candidates, of which 45 are in clinical trials and five have received interim or conditional approval for emergency use in different countries. These include the traditional inactivated and attenuated vaccines, as well as novel DNA- and RNA-based vaccines. Major advantages and limitations of each vaccine type have been discussed in detail elsewhere (Badgujar et al., 2020; Rawat et al., 2020). Among these, five have demonstrated promising results in preliminary phase III trials (Table 2). AZD1222 (formerly ChAdOx1-S, from Oxford/AstraZeneca) is a non-replicating viral vector vaccine comprising the SARS-CoV-2 spike protein that has demonstrated protective effects in rhesus macaques after a single dose (Van Doremalen et al., 2020), and 62–90% efficacy when given in two-dose regimen in humans (Voysey et al., 2020). Vaccines developed by Moderna and Pfizer/BioNTech (mRNA-based vaccines) (Jackson et al., 2020; Polack et al., 2020), Gamaleya (recombinant viral vector-based vaccine) (Logunov et al., 2020), and Sinovac (inactivated viral-based vaccine) (Zhang Y. et al., 2020) were also reported to have high efficacy. The importance of vaccine development notwithstanding, alternative therapeutic approaches are being concomitantly investigated to identify effective therapies against COVID-19 with the necessary urgency.

**TABLE 2:** Vaccine candidates with promising efficacy in phase III trials.

Company	Type	Doses required	Efficacy reported in preliminary or interim analyses of phase III trials
Gamaleya	Two different adenoviral vectors (Ad26 and Ad5)	2×	∼90%
Moderna	mRNA-based (mRNA-1273)	2×	∼94%
Oxford/AstraZeneca	Adenovirus-based (ChAdOx1)	2×	62-90%
Pfizer/BioNTech	mRNA-based (BNT162b2)	2×	∼95%
Sinovac	Inactivated viral-based	2×	50–80%

### Therapeutic Approaches Aimed at Preventing Cell Entry and Replication of SARS-CoV-2

#### TMPRSS2 Inhibitors

Camostat mesilate (Foipan^TM^) is a serine protease inhibitor clinically approved for squamous cell carcinoma and chronic pancreatitis in Japan. It significantly reduces viral load of SARS-CoV, human coronavirus NL63, and SARS-CoV-2 *in vitro* by partially blocking TMPRSS2 activity (Kawase et al., 2012; Hoffmann et al., 2020a). Nafamostat mesilate (Buipel^TM^) is another serine protease inhibitor used in Japan that has been demonstrated to inhibit MERS-CoV and SARS-CoV-2 entry into host cells by targeting TMPRSS2 (Hoffmann et al., 2020b; Yamamoto et al., 2020). Nafamostat was even more effective at inhibiting SARS-CoV-2 infection *in vitro* than camostat (Yamamoto et al., 2016, 2020; Hoffmann et al., 2020b). Furthermore, nafamostat is approved for disseminated intravascular coagulation in Japan due to its anticoagulant properties, which is an additional advantage for the treatment of COVID-19, given the high prevalence of coagulation disturbances described above (Tang et al., 2020; Zhou F. et al., 2020).

#### Cathepsin Inhibitors

*In vitro* studies demonstrated that E64d, a non-selective cysteinyl cathepsin inhibitor, was able to limit both SARS-CoV and SARS-CoV-2 infection in human epithelial cells, while the combination of E64d with a TMPRSS2 inhibitor completely abrogated viral entry (Hoffmann et al., 2020a, b). The investigational compound K11777 and three of its analogs demonstrated strong antiviral activity against SARS-CoV pseudotypes *in vitro* (Zhou et al., 2015). Oxocarbazate was also effective at inhibiting SARS-CoV and Ebola virus entry into cells (Shah et al., 2010). These agents, all cathepsin inhibitors, have potential therapeutic utility in COVID-19.

#### RAAS Modulators

Some concerns have been raised regarding the long-term use of ACE inhibitors or angiotensin receptor blockers (e.g., captopril, losartan) for individuals with pre-existing cardiovascular diseases during the COVID-19 pandemic, as these drugs might upregulate ACE2 and could theoretically enhance susceptibility to SARS-CoV-2 infection and COVID-19 severity. However, multiple studies have found no correlation between use of RAAS inhibitors and likelihood of testing positive for SARS-CoV-2 infection, nor with COVID-19 severity in those infected (Iaccarino et al., 2020; Khera et al., 2020; Mancia et al., 2020; Reynolds et al., 2020). In fact, in a retrospective study, a reduction in COVID-19–related mortality was observed in hospitalized individuals with hypertension who had been treated with ACE inhibitors or angiotensin receptor blockers compared to those not using any of these drugs (Zhang P. et al., 2020). Furthermore, abrupt discontinuation of RAAS inhibitors in individuals with cardiovascular disease and on long-term therapy is not recommended, as it may cause clinical decompensation (Danser et al., 2020). Based on the protective role of ACE2 as a counter-regulator of Ang II/AT1-R effects, therapeutic approaches that restore the balance between ACE and ACE2 would be ideal to mitigate COVID-19–induced multiple organ injury in individuals without pre-existing medical conditions, preferably in combination with an effective antiviral agent.

#### Recombinant Soluble ACE2 and Human Anti-SARS-CoV-2 Monoclonal Antibodies

It has been proposed that a recombinant soluble form of ACE2 (rhACE2), administered exogenously, may competitively bind to the SARS-CoV-2 RBD and thus prevent its interaction with native ACE2 for host-cell entry. Such an approach not only would neutralize the virus but also preserve ACE2 activity, thus protecting lung and cardiovascular tissues from injury (Monteil et al., 2020). In a pilot study, one rhACE2 formulation (known as GSK2586881) demonstrated no safety concerns and was able to decrease levels of Ang II with a trend toward decreasing IL-6 levels in serum (Kahn et al., 2017). This rhACE2 also inhibited SARS-CoV-2 infection in both engineered human blood vessels and kidney organoids *in vitro* (Monteil et al., 2020). A clinical trial is ongoing to evaluate the effects of rhACE2 in individuals with COVID-19 (NCT04335136). Alternatively, CR3022 is a SARS-CoV-specific human monoclonal antibody that was able to bind to SARS-CoV-2 RBD and neutralize viral actions. CR3022 may be a promising candidate to prevent or significantly reduce SARS-CoV-2 infection (Tian X. et al., 2020). A monoclonal antibody obtained from B-cells of individuals recently recovered from COVID-19 has also demonstrated ability to inhibit SARS-CoV-2 RBD and ACE2 interactions (Chen X. et al., 2020).

#### Antiviral Drugs

An ideal antiviral agent should target factors that are highly conserved among coronaviruses and essential for viral pathogenesis. A number of antivirals under investigation for COVID-19 are nucleoside analogs used for other viruses. These molecules are incorporated into nascent DNA/RNA chains during viral replication, and lead to premature termination of nucleic acid synthesis or insertion of mutations in the viral genome that prevent subsequent viral replication. Ribavirin is a guanosine analog used for the treatment of hepatitis C virus, respiratory syncytial virus, and certain viral hemorrhagic fevers. A recent *in vitro* study demonstrated that high-dose ribavirin can reduce SARS-CoV-2 infection (Wang M. et al., 2020). It was also tested with and without IFN-α in individuals infected with SARS-CoV and MERS-CoV, but whether it has any therapeutic benefit remains controversial (Zhao et al., 2003; Arabi et al., 2020). Severe adverse effects were also reported, including hepatotoxicity and hemolysis.

Favipiravir (Avigan^TM^) is another guanosine analog that selectively inhibits the influenza viral RNA-dependent RNA polymerase (RdRp). It has been approved against influenza virus but also demonstrated antiviral activity against Ebola virus, yellow fever virus, and other viruses in experimental models (Furuta et al., 2017). As with ribavirin, a high dose of favipiravir was able to reduce SARS-CoV-2 infection *in vitro* (Wang M. et al., 2020). In an early clinical study, favipiravir demonstrated better clinical outcomes compared to umifenovir (see below), with a significant decrease in fever and cough in individuals with COVID-19 (Chen N. et al., 2020). In a similar study, favipiravir therapy was associated with a shorter time to viral clearance and higher improvement rate in chest imaging of individuals with COVID-19 (Cai et al., 2020), resulting in extension of its approved indications by the National Medical Products Administration of China.

Remdesivir (Veklury^TM^) is an adenosine analog with a broad spectrum of antiviral activity against several RNA viruses. It blocks RdRp, thus preventing an early step of viral replication (Gordon C.J. et al., 2020; Sheahan et al., 2020). Remdesivir has been shown to inhibit viral replication of Ebola virus, MERS-CoV, and SARS-CoV-2 in experimental studies, with higher efficacy compared to other antiviral agents (Gordon C.J. et al., 2020; Sheahan et al., 2020; Wang M. et al., 2020). Furthermore, it demonstrated a favorable benefit-risk profile compared to placebo in early-stage clinical study in individuals with severe COVID-19 (Davies et al., 2020). In a compassionate-use setting, remdesivir was used in hospitalized individuals with severe COVID-19 and 68% of the cohort demonstrated an improvement in oxygen-support class and 25% were discharged during 18 days follow-up (Grein et al., 2020). In phase III multicenter randomized clinical trials, remdesivir appeared to shorten the recovery time of some patients, although therapeutic benefits were not clearly demonstrated in terms of reducing fatality rate of individuals with severe COVID-19 (Beigel et al., 2020; Wang et al., 2020b). Despite its inefficiency in improving survival (Dyer, 2020), final reports of the multicenter clinical trial indicated that remdesivir might shorten the time to recovery of hospitalized adults with COVID-19 and reduce lower respiratory tract infection, which culminated in its FDA approval.

Besides nucleoside analogs, antiviral agents may block viral replication through distinct mechanisms, including inhibition of endosomal acidification and inhibition of proteases essential for intracellular assembly. The fixed-dose combination lopinavir/ritonavir (Kaletra^TM^) is an FDA-approved therapy for human immunodeficiency virus (HIV). Lopinavir is an inhibitor of HIV-1 protease, while ritonavir prevents the rapid metabolism of lopinavir by inhibiting CYP3A isoenzymes. This combination has demonstrated antiviral activity against SARS-CoV and MERS-CoV, reducing viral load in experimental studies (Chu et al., 2004; Chan et al., 2015), and was associated with modest clinical improvement of MERS in a case report (Kim et al., 2016). Furthermore, a milder disease course was observed in individuals infected with SARS-CoV who received lopinavir/ritonavir (Chu et al., 2004). Initially, lopinavir/ritonavir appeared to accelerate the recovery process from COVID-19, shortening ICU length of stay compared to standard therapy alone; however, it has since proven unable to reduce viral load or mortality in adults hospitalized with severe COVID-19 (Cao et al., 2020; Osborne et al., 2020). Darunavir is a second-generation protease inhibitor used in combination with ritonavir or cobicistat for HIV therapy. It was also considered as a potential therapeutic alternative in COVID-19; however, darunavir demonstrated no antiviral activity against SARS-CoV-2 *in vitro* (De Meyer et al., 2020).

Umifenovir (Arbidol^TM^) is a dual-acting antiviral/host-targeting agent approved for treatment of influenza in Russia and China. It inhibits membrane fusion of viral envelope and host cell by preventing clathrin-mediated endocytosis and has been demonstrated to prevent *in vitro* infection with several common pathogenic viruses (Pécheur et al., 2016). In an early clinical study of COVID-19, umifenovir therapy was associated with a trend toward reductions in viral load and mortality rate, albeit not statistically significant (Wang Z. et al., 2020). In a retrospective study, most umifenovir-treated individuals demonstrated SARS-CoV-2 negativity and improvement in chest imaging after 14 days of therapy (Deng et al., 2020). However, there is no additional evidence to support that umifenovir may improve clinical outcomes of individuals with COVID-19.

Two investigational antiviral drug candidates (11a and 11b) have been developed by structure-based design methods to target 3CL protease (M^pro^), the main SARS-CoV-2 protease. Both demonstrated good pharmacokinetic properties and low toxicity and were able to significantly reduce viral load *in vitro* (Dai et al., 2020). Further development is ongoing.

Despite some promising results in experimental and early clinical studies, large-scale randomized trials are still in progress and their results on safety and efficacy will provide a better guidance for the potential use of these drugs in the treatment of COVID-19.

#### Other Anti-infectives

Ivermectin and nitazoxanide are two clinically approved antiparasitic agents that have demonstrated significant antiviral activity against SARS-CoV-2 infection *in vitro* (Caly et al., 2020; Rocco et al., 2020; Wang Z. et al., 2020). A recent study (Rocco et al., 2020) confirmed that early administration of nitazoxanide (1-3 days after onset of symptoms) reduced the viral load in individuals with mild COVID-19 with a good safety profile, even though more studies are required to evaluate its efficacy in individuals with both mild and severe COVID-19. Clinical safety and efficacy of ivermectin in COVID-19 have yet to be confirmed.

Chloroquine and hydroxychloroquine are aminoquinoline antimalarials also used in the treatment of several autoimmune diseases. Both drugs have demonstrated a broad spectrum of antiviral activity *in vitro*—including against coronaviruses—through various mechanisms, such as blocking ACE2 terminal glycosylation and hindering endosome–lysosome fusion (De Wilde et al., 2014; Mauthe et al., 2018; Wang M. et al., 2020). In early clinical studies, chloroquine and hydroxychloroquine were suggested to reduce viral load and improve clinical outcomes in individuals with COVID-19 (Gao et al., 2020; Gautret et al., 2020) with an even better effect when combined with azithromycin (Gautret et al., 2020). However, such findings were not confirmed in later, well-designed clinical trials; both chloroquine and its hydroxy analog appear ineffective, whether used therapeutically or for postexposure prophylaxis, with conflicting evidence regarding their safety and toxicity (Borba et al., 2020; Boulware et al., 2020; Cavalcanti et al., 2020; Geleris et al., 2020; Lane et al., 2020; Molina et al., 2020).

#### Convalescent Plasma Therapy

Convalescent plasma (CP) is plasma rich in neutralizing antibodies which has been extracted from individuals who have recovered from an infection. This plasma is processed and then administered to infected individuals. CP has been demonstrated to reduce viral load and improve clinical outcomes in other coronavirus infections, specifically SARS and MERS (Cheng et al., 2005b; Ko et al., 2018). In early-stage clinical studies, CP therapy was well tolerated and improved clinical outcomes by neutralizing viremia in severe COVID-19 (Duan et al., 2020; Shen et al., 2020; Ye et al., 2020). Improvements in clinical scales as well as discharge and survival rate in individuals with severe COVID-19 were observed 14 days after CP therapy (Salazar et al., 2020; Zhang et al., 2020a). Several clinical trials of CP are in progress. Based on previous experience with influenza A (H1N1), some practical limitations may apply, and should be borne in mind when seeking to implement an effective CP therapy regimen for COVID-19. These limitations have been discussed elsewhere (Wong et al., 2010). Antibodies produced in humanized mouse or equine serum have been investigated as alternative strategies to neutralized SARS-CoV-2 with some promising results – up to 100 times more potent than convalescent plasma from COVID-19-recovered individuals (Cunha et al., 2020; Hansen et al., 2020).

### Therapeutic Approaches Aimed at Immunomodulation and Tissue Repair

#### IL-6 Inhibitors

IL-6 is acutely induced by inflammatory stimuli and mediates a number of immune responses. Individuals with COVID-19 demonstrate an increase in serum levels of IL-6 within 3 days after disease onset, with even higher levels in severe cases compared to mild ones (Liu J. et al., 2020). A further increase in IL-6 levels has also been associated with increased risk of respiratory failure and death (Herold et al., 2020; Quartuccio et al., 2020; Ruan et al., 2020).

Tocilizumab (Actemra^TM^) is a recombinant humanized monoclonal antibody that binds and inhibits IL-6 receptor activity. It is indicated for the treatment of autoimmune disorders and cytokine release syndrome and has been suggested as a potential therapeutic option for COVID-19-induced hyperinflammation (Zhang et al., 2020a). In early clinical studies, tocilizumab significantly improved several outcomes in severe and critical COVID-19 cases, including supplemental oxygen utilization and C-reactive protein and D-dimer levels (Sciascia et al., 2020; Xu X. et al., 2020). In a subsequent study, tocilizumab therapy was associated with clinical improvements, as well as rapid and sustained benefits in ICU and non-ICU patients with COVID-19 (Toniati et al., 2020). Alternatively, siltuximab (Sylvant^TM^) is a chimeric monoclonal antibody that directly bind to IL-6. Siltuximab provides an advantage compared to tocilizumab as it neutralizes circulating IL-6, which could contribute to neurotoxic effects. In an early clinical study, siltuximab therapy at the onset of mechanical ventilation was associated with reduced occurrence of respiratory failure and death in severe COVID-19 cases (Gritti et al., 2020). Sarilumab is another IL-6R inhibitor that appears to be a potential therapy against severe COVID-19 (Gremese et al., 2020).

#### IL-1 Inhibitors

The ORF3a protein of coronaviruses can activate NF-κB signaling and the NLRP3 inflammasome. The inflammasome activates cleavage of pro-IL-1β by caspase-1 into active IL-1β, which mediates lung inflammation and fibrosis (Siu et al., 2019). Anakinra (Kineret^TM^), a recombinant IL-1 receptor antagonist, was evaluated in individuals with severe COVID-19 and demonstrated effective reductions in need for mechanical ventilation and fatality rate in an early clinical study (Huet et al., 2020). A retrospective study also indicated that high-dose anakinra was safe and associated with clinical improvements in over 70% of individuals with severe COVID-19 (Cavalli et al., 2020). At least 10 additional clinical trials are ongoing with the aim of evaluating the effects of anakinra in targeting hyperinflammation in COVID-19 (King et al., 2020). Colchicine is another drug known to reduce neutrophil recruitment and IL-1β levels, and widely used for the treatment of gout. In hospitalized individuals with COVID-19, colchicine did not affect C-reactive protein or cardiac troponin levels, although an improved time to clinical deterioration was reported (Deftereos et al., 2020).

#### Janus Kinase/Signal Transducer and Activators of Transcription (JAK/STAT) Inhibitors

Targeting the JAK/STAT pathway appears to be a promising approach, given its role in cytokine receptors on immune cells. JAK/STAT inhibitors have also been used for the treatment of cytokine release syndrome. Several clinical trials evaluating the effects of JAK/STAT inhibitors for COVID-19 are ongoing. Fedratinib, a JAK2 inhibitor, has been hypothesized to inhibit SARS-CoV-2 and Th17-induced inflammation without modulating IFN signaling, but efficacy remains to be investigated (Wu and Yang, 2020). In a recent clinical study of individuals with COVID-19, ruxolitinib, a JAK1/2 inhibitor, was not significantly superior to placebo in terms of clinical parameters, although faster recovery from lymphopenia was observed (Cao et al., 2020b). On the other hand, baricitinib, a high-affinity JAK1/2 inhibitor, has been shown to improve some clinical and laboratory parameters (including C-reactive protein levels) in a pilot study of COVID-19 cases (Cantini et al., 2020).

#### Corticosteroids

There is some controversy regarding corticosteroid therapy in COVID-19 (Mattos-Silva et al., 2020). Although corticosteroids are widely used to suppress lung inflammation, they were associated with delayed viral clearance and no improvement in fatality rate during the SARS and MERS epidemics (Li et al., 2020a). Nevertheless, methylprednisolone therapy has been shown to improve chest imaging, reduce fatality rate, and shorten hospital stay in individuals with severe COVID-19 (Ramiro et al., 2020; Wang et al., 2020a).

Recent preliminary results of low-dose dexamethasone therapy also demonstrated a significant reduction in fatality rate (up to one-third) in critically ill individuals with COVID-19, although such benefits were not observed for the cohort of individuals who did not require oxygen therapy at admission (Horby et al., 2020). It is possible that corticosteroid therapy may be beneficial in certain phases of COVID-19, such as the hyperinflammatory stage, but certainly should be combined with an effective antiviral or antibiotic agent to reduce the risk of superinfection. Further clinical studies are necessary to better understand the effects of corticosteroid therapy in COVID-19.

#### Adjuvant Therapy

Unfractionated or low-molecular-weight heparin has been used prophylactically in hospitalized individuals with COVID-19 to prevent the occurrence of thromboembolic events. Heparin can also induce immunomodulatory effects and protect endothelial cells from oxidative stress, thus preventing increased vascular permeability, microthrombus formation, and leukocyte extravasation. In an observational study, anticoagulant therapy with low-molecular-weight heparin was associated with better prognosis in severe COVID-19 cases with high levels of D-dimer (Tang et al., 2020). Despite current recommendations for the prophylactic use of low-molecular-weight heparin in all hospitalized COVID-19 patients (except those with contraindications) (Thachil et al., 2020), individuals admitted to the ICU remain at high risk of pulmonary embolism (Klok et al., 2020).

The use of recombinant human DNase (dornase) to disrupt NETs has been proposed, as SARS-CoV-2 was found to induce excessive production of NETs, which may contribute to thromboembolism events, cytokine storm, and tissue injury (Barnes et al., 2020; Veras et al., 2020). Dornase alfa (Pulmozyme^TM^) has been long used as an inhalation solution for mucus clearance in individuals with cystic fibrosis; however, its effects on COVID-19 remain to be investigated. Bevacizumab (Avastin^TM^/Zaribev^TM^) is an anti-VEGF humanized monoclonal antibody that has been investigated to reduce lung edema in COVID-19 (Samudrala et al., 2020). Finally, mepolizumab (Nucala^TM^), an anti-CD147 humanized antibody used for the treatment of severe asthma, demonstrated to improve the recovery of individuals with COVID-19-induced severe pneumonia in a small-scale clinical study (Bian et al., 2020).

#### Mesenchymal Stromal Cell (MSC)-Based Therapies

Mesenchymal stromal cells and their biologically active products, such as extracellular vesicles, are known to induce immunomodulatory and reparative effects, reducing lung and distal organ injury and improving survival in several preclinical models of ARDS and sepsis (Matthay et al., 2017; Silva et al., 2018, 2019; Lopes-Pacheco et al., 2020). In early clinical studies, MSC administration was well tolerated and caused no obvious safety concerns in critically ill individuals (McIntyre et al., 2018; Matthay et al., 2019; Khoury et al., 2020). Notably, SARS-CoV-2 is unable to infect MSCs, as these cells do not express ACE2 (Leng et al., 2020). In one case report of compassionate use in severe COVID-19, administration of Wharton’s jelly-derived MSCs reduced plasma levels of IL-6, TNF-α, and C-reactive protein and improved lung function (Zhang Y. et al., 2020). In another early clinical study, bone marrow-derived MSCs were administered intravenously to seven individuals with severe COVID-19. MSC administration was safe and significantly reduced inflammation, resulting in improvements in symptoms and lung function (Leng et al., 2020). Alternatively, administration of bone marrow MSC-derived exosomes was evaluated in an early clinical study of individuals with severe COVID-19 and demonstrated no adverse events (Sengupta et al., 2020). Several other clinical trials are evaluating MSC-based therapies for COVID-19. Despite the great promise of MSCs for the treatment of COVID-19 complications, such as ARDS and sepsis, several open questions remain, including the best source, dose, route of administration, frequency, and timing (De Castro et al., 2019; Khoury et al., 2020; Lopes-Pacheco et al., 2020). Further understanding of the effects of MSCs on COVID-19 pathogenesis and their underlying mechanisms are also needed in order to translate MSC-based therapy into clinical practice with safety and effectiveness.

## Outlook and Considerations

COVID-19 can cause not only severe lung injury but also multiple organ dysfunction with potential long-term effects on survivors. The downregulation of membrane-bound active ACE2 induced by SARS-CoV-2 infection can be detrimental to everyone, but is particular so for individuals whose baseline ACE2 expression is already deficient. Although infected individuals with certain pre-existing medical conditions are more prone to developing severe COVID-19, the disease is not exclusively restricted to this population, and a combination of multiple direct and indirect pathogenic factors contribute to disease severity and a broad spectrum of phenotypes. Even though vaccination has initiated in multiple countries, the production of vaccines on a global scale will take some time, despite having different vaccines approved against SARS-CoV-2. Accordingly, implementation of preventive measures remains crucial to limit rapid dissemination of the virus and potential COVID-19-induced organ injuries, as well as prevent saturation of national healthcare systems.

It is imperative to continuously monitor genetic modifications of coronaviruses, as any gain-of-function mutation affecting the life-cycle pathways of SARS-CoV-2 (and other viruses in this family) can make them more infectious and lethal to humans. In this context, a SARS-CoV-2 subtype with the D614G mutation in the spike protein was recently found to confer increased infectivity (Bhattacharyya et al., 2020; Hou et al., 2020); further investigations are ongoing to elucidate the lethality rate of this and other subtypes. More strict regulations against the trade of wild animals for domestication or food should also be implemented to prevent potential future pandemics.

Over the last two decades, we have witnessed three coronavirus outbreaks. The unprecedented consequences of COVID-19 pandemic have prompted a massive global research effort to better understand the pathological mechanisms underlying SARS-CoV-2 infection and—at an accelerated pace—develop safe and efficient therapies against this devastating condition. Although a number of these therapies have demonstrated promising results in early clinical studies, safety and efficacy remain to be demonstrated in large-scale clinical trials. A greater understanding of the clinical features has provided better guidance in managing the disease to prevent further complications. Furthermore, the phenotypical differences in COVID-19 manifestations suggest that personalized therapeutic regimens should be considered on a case-by-case basis, ranging from prevention of viral cell entry and replication to supportive, immunomodulatory and tissue reparative approaches. Therefore, multi-target therapeutic protocols may be the best option to achieve a higher number of individuals with severe COVID-19 and significantly reduce or even prevent multiple organ dysfunction.

## Author Contributions

ML-P contributed to the design and conceptualization, original draft, editing, and review for intellectual content. PS, FC, DB, CR, PP, MM, and CCN reviewed the intellectual content. PR did the conceptualization and edited and reviewed the intellectual content. All authors read and approved the final version of the manuscript.

## Conflict of Interest

The authors declare that the research was conducted in the absence of any commercial or financial relationships that could be construed as a potential conflict of interest.

## Footnotes

1 WHO Director-General’s opening remarks at the media briefing on COVID-19 – 11 March 2020: https://www.who.int/dg/speeches/detail/who-director-general-s-opening-remarks-at-the-media-briefing-on-covid-19—11-march-2020

2 WHO Coronavirus Disease (COVID-19) Dashboard: https://covid19.who.int/

3 Johns Hopkins University & Medicine – Coronavirus Resource Center: https://coronavirus.jhu.edu/map.html

4 http://clinicaltrials.gov
